# Application of multiomics analysis to plant flooding response

**DOI:** 10.3389/fpls.2024.1389379

**Published:** 2024-08-13

**Authors:** Guangya Gui, Qi Zhang, Weiming Hu, Fen Liu

**Affiliations:** ^1^ College of Traditional Chinese Medicine, Jiangxi University of Chinese Medicine, Nanchang, China; ^2^ Lushan Botanical Garden, Jiangxi Province and Chinese Academy of Sciences, Jiujiang, China

**Keywords:** flooding, waterlogging, submergence, hypoxia, metabolomics, proteomics, transcriptomics

## Abstract

Flooding, as a natural disaster, plays a pivotal role in constraining the growth and development of plants. Flooding stress, including submergence and waterlogging, not only induces oxygen, light, and nutrient deprivation, but also alters soil properties through prolonged inundation, further impeding plant growth and development. However, hypoxia (or anoxia) is the most serious and direct damage to plants caused by flooding. Moreover, flooding disrupts the structural integrity of plant cell walls and compromises endoplasmic reticulum functionality, while hindering nutrient absorption and shifting metabolic processes from normal aerobic respiration to anaerobic respiration. It can be asserted that flooding exerts comprehensive effects on plants encompassing phenotypic changes, transcriptional alterations, protein dynamics, and metabolic shifts. To adapt to flooding environments, plants employ corresponding adaptive mechanisms at the phenotypic level while modulating transcriptomic profiles, proteomic characteristics, and metabolite levels. Hence, this study provides a comprehensive analysis of transcriptomic, proteomic, and metabolomics investigations conducted on flooding stress on model plants and major crops, elucidating their response mechanisms from diverse omics perspectives.

## 1 Introduction

Flooding stress is a prominent abiotic stress factor throughout the plant life cycle (Jackson and Colmer, 2005; Manghwar et al., 2024). There are two primary types of flooding stress: (1) “waterlogging,” which refers to the inundation of plant roots; (2) submergence, where the entire plant is submerged in water (Sasidharan et al., 2017). In both cases, flooding can impede seedling growth and reduce crop yields, posing a significant threat to plant growth and productivity (Komatsu et al., 2013; Bailey-Serres and Colmer, 2014). Firstly, flooding alters soil properties, leading to an increase in soil pH value. The concentrations of micronutrients, Zn and Cu, decrease over time, while macronutrients, K, Mg, and Ca, exhibit higher levels during early stages of flooding, but gradually decline later. Additionally, toxic element, As, increases with prolonged flooding duration (Bailey-Serres and Colmer, 2014; Wang et al., 2020). This change in environmental conditions makes most plants and major crops highly susceptible to flooding, except for rice and certain herbage plants (Sasidharan et al., 2018; Klaas et al., 2019; Fukushima et al., 2020; Wang et al., 2021a). In addition, flooding leads to oxygen deprivation in the environment, thereby inducing reactive oxygen species (ROS) production in plant mitochondria. Plants can adapt to flooding stress by regulating their growth and development through ROS signaling pathways, such as adventitious root (AR) formation and stomatal opening (Pucciariello and Perata, 2021). Simultaneously, plants possess a comprehensive ROS clearance system, comprising enzymatic and non-enzymatic pathways, that effectively counteract oxidative stress caused by flooding. ROS not only directly participate in plant responses to flooding stress, but also interact with other signaling molecules to collectively regulate plant stress responses. Furthermore, flooding significantly impedes gas exchange, retarding the rate of gas diffusion and adversely affecting photosynthesis and respiration (Herzog et al., 2018; Nadarajah, 2020). Some muddy flooding can almost completely block light penetration, further hampering photosynthesis. Consequently, insufficient internal energy supply leads to severe stunting. When flooding recedes, previously adapted plant tissues abruptly exposed from low-light oxygen-deprived environments to aerial conditions, with intense light exposure damage and reoxygenation stresses occur (Yeung et al., 2018). Thus, flooding represents continuous stressors that impact plants differently at various stages.

Plants employ two strategies to cope with flooding (Voesenek and Bailey-Serres, 2013; Bashar et al., 2019; Xu et al., 2023): the first strategy is escape, whereby plants actively respond to the gas exchange between submerged organs and the aerial environment by augmenting energy production, preferentially stimulating stem or leaf growth, and subsequently establishing adventitious roots to enhance root porosity. This strategy enables plants to acquire more oxygen and extend their survival time in water (Wu et al., 2022). The second strategy is quiescence, where plants respond negatively by suppressing anaerobic respiration to a lower level, reducing unnecessary energy metabolism, and prolonging underwater survival through energy conservation and carbohydrate preservation (Zhou et al., 2020). These strategies are exemplified by different genotypes of pea seeds during germination under waterlogging conditions. Some seeds adopt tolerance strategies by up-regulating tyrosine protein kinase while down-regulating fat metabolism gene linoleate 9S-lipoxygenase 5 (LOX5). On the other hand, some seeds employ an escape strategy through upregulation of subtilaze family proteins and fat metabolism gene peroxisomal adenine nucleotide carrier 2 (PNC2) (Zaman et al., 2019). Certain waterlogging-resistant plant varieties comprehensively utilize both these strategies. For instance, rice, wheat, and corn shift from aerobic respiration to glycolysis and fermentation via AR formation and root aerenchyma development. They adapt by adjusting traits and altering energy supply mechanisms, and using antioxidant enzymes that scavenge ROS in waterlogging environments (Bailey-Serres et al., 2012; Shah et al., 2018; Rafique, 2019; Wang et al., 2020). Different plant tissues exhibit distinct responses to anoxia induced by flooding. In wheat, for example, roots and coleoptiles shown different metabolic adaptation to anoxia (Huang et al., 2018).

In recent years, with the rapid advancement of omics technology, an increasing number of researchers have embraced its application in the investigation of plant flooding responses. Omics technology enables a comprehensive analysis of plants, thus facilitates profound exploration into the ecological impact of flooding disasters and the adaptive mechanisms employed by plants within flooding environments. Metabolomics primarily focuses on the identification and quantification of plant metabolites, closely associated with phenotypes. Metabolomics analysis by mass spectrometry technology is extensively employed as a highly specific analytical technique to gain insights into plant processes, revealing the physiological and biochemical status of plant cells under normal or stress conditions. Investigating the levels and types of metabolites in plants subjected to flooding stress can elucidate their response mechanisms (Abdelrahman et al., 2018). Additionally, plants modulate metabolism by altering protein abundance. Proteomics enables comparison and analysis of protein abundance between flooding-tolerant and -intolerant plants, facilitating comprehension of functional changes in proteins during flooding conditions, while unraveling regulatory mechanisms (Komatsu et al., 2012). Transcriptomics can be utilized to infer the potential of key regulatory factors and their targets in plant gene regulatory networks on flooding stress, elucidating the underlying mechanisms driving these changes in plants. Plants undergo intricate morphological and physiological adaptations to abiotic stresses, such as flooding, necessitating complex reorganization of transcriptional and post-transcriptional controlled gene expression networks. In addition, microRNAs (miRNAs) and transcription factors (TFs) also play pivotal roles in this process (Liu et al., 2012). Initially, TFs bind to promoter regions to modulate the transcription of specific transcripts, while miRNAs regulate the expression of their target transcripts through cleavage or translation inhibition (Sharma et al., 2020). TFs interact with miRNAs at both transcriptional and post-transcriptional levels, exerting significant influence on plant growth and development under stress conditions (Li and Zhang, 2016).

Therefore, this paper aims to provide a comprehensive understanding of the molecular, cellular, physiological, and biochemical responses of model plants and major crops to flooding stress by summarizing their common points and similarities. Herein, we listed the transcriptomic, proteomic, and metabolomics results obtained from these plants, to elucidate potential mechanisms and adaptive strategies employed by them in response to flooding stress (refer to **Supplementary Table 1** for a summary).

## 2 Transcriptomics can help elucidate the regulatory mechanisms of flooding resistance in plants

Transcriptomics investigates gene transcription and transcriptional regulation in cells at a global level, examining gene expression at the RNA level. The standard batch RNA-Seq technique is applied to each transcript, providing an average expression level sample encompassing various cell types. Transcriptome profiles offer insights into genes expressed under specific conditions, infer the functions of unknown genes, and unveil regulatory mechanisms of action for specific genes. By establishing differential expression profiles from transcriptomes, detailed descriptions can be provided regarding biological responses to environmental changes and other factors (Li et al., 2018a).

Environmental alterations caused by flooding can impact metabolic, physiological, and developmental processes in plants due to specific modifications in transcription and translation events (Yu et al., 2020; Li et al., 2022). For instance, waterlogging-induced exposure significantly alters the expression patterns of transcription factors such as Basic leucine zipper (bZIP) gene family members ethylene response factors (ERFs), MYBs, bHLHs, and WRKYs, compared to normal conditions in sesame (Wang et al., 2016, 2018; Dossa et al., 2019). A set of miRNA were identified from small RNA-Seq of Maize under waterlogging, and functional analysis suggested that miRNA active participants in defense of waterlogging (Liu et al., 2012). It is evident that comparing transcriptome data can reveal differences in gene expression in plants under flooding conditions. Further analysis of the function and regulatory relationship of these differentially expressed genes can provide insights into the regulatory mechanism of plant response to flooding.

### 2.1 Activation of genes involved in glucose metabolism provides energy

The primary consequence of flooding is the exposure of plants to an oxygen-deprived environment. In response to anoxic conditions, plants promptly enhance carbohydrate metabolism through glycolysis for energy production (Zou et al., 2010; Butsayawarapat et al., 2019; Kaur et al., 2021). Waterlogging-induced up-regulation of PDC, alcohol dehydrogenase (ADH), and sucrose synthase (SUS) genes, which are associated with sucrose synthase, glycolysis, and fermentation pathways. However, after the waterlogging waters receded, the expression levels of these genes decreased (Zhao et al., 2018). Furthermore, the activity of glycolytic enzymes is also affected. Hypoxia induces a shift in sugar metabolism and inhibits the tricarboxylic acid cycle, leading to increased glycolytic flux and accumulation of succinate in grapevine roots (Ruperti et al., 2019). A similar response is observed in cucumbers and cruciferous species. Transcriptomic analysis of root systems from various species revealed that flooding induces the activation of genes associated with glycolysis, facilitating energy release under different durations of inundation. Additionally, we noted a positive correlation between early upregulation of glycolytic genes and enhanced flooding tolerance. Conversely, varieties exhibiting poor waterlogging tolerance exhibited delayed regulation of glycolysis-related genes (Hwang et al., 2020; Kęska et al., 2021).

### 2.2 Flooding changes the expression of amino acid-related genes

Amino acid metabolism plays a pivotal role in response to flooding stress. Under waterlogging conditions, cucumber root system regulates gene expression related to amino acids, including the degradation pathways of valine, leucine, and isoleucine. Concurrently, lactic acid accumulation increases during hypoxic stress caused by waterlogging, resulting in a decrease in cytoplasmic pH. Aspartate transferase and glutamate decarboxylase, integral components of amino acid metabolism, play a crucial role in regulating the cytoplasmic pH value of cucumber plants (Kęska et al., 2021). Grape roots exposed to low oxygen levels caused by flooding exhibit overexpression of numerous genes involved in amino acid metabolism (Ruperti et al., 2019). Furthermore, waterlogging-induced stress upregulates glutathione peroxidase (GPX) and glutathione S-transferases (GST) genes in alfalfa plants, thereby activating pathways associated with amino acid metabolism such as amino sugar and nucleotide sugar metabolism as well as arginine and proline metabolism (Zeng et al., 2019).

### 2.3 ROS related factors are regulated by flooding

Flooding can significantly increase the level of ROS in plants and destroy the homeostasis of ROS. In response to damages caused by ROS, plants promptly upregulate genes encoding ROS scavenger enzymes, like catalase, to mitigate ROS levels early while safeguarding cells from death (Raza et al., 2021). Furthermore, phenylpropanoid biosynthesis pathway, which plays a crucial role in tolerance to various oxidative stresses in plants, was notably enriched in waterlogging-resistant varieties of the same plant species under waterlogging stress (Wang et al., 2021a). The expression of transcription factors and genes associated with the ROS signaling pathway was found to be lower in less tolerant varieties, suggesting that enhancing ROS scavenging enzyme activity and reducing ROS accumulation could potentially enhance plant tolerance to waterlogging stress. Additionally, increased ROS scavenging enzyme activity during reoxygenation after waterlogging has been observed in both monocotyledonous and dicotyledonous plants, aiding their ability to cope with reoxygenation-induced damage and adapt to changing environmental conditions (Yuan et al., 2017; Zhao et al., 2018; Yu et al., 2020).

### 2.4 Genes involved in metabolite synthesis are also regulated by flooding

Meanwhile, the synthesis of specific secondary metabolites, such as lipids and hormones, significantly contributes to plant stress response, including flooding. For instance, in cucumber, esterase/lipase genes were strongly upregulated under waterlogging, suggested enhanced lipid metabolism with tolerance to waterlogging (Kęska et al., 2021).

The expression of transcription factors associated with hormonal responses, particularly ethylene, exhibited significant alterations during flooding stress. Ethylene is a key hormone in plant germination, differentiation, flowering and fruiting (Dubois et al., 2018), and oxygen induction is controlled by ethylene signaling under hypoxic conditions. The ethylene precursor 1-aminocyclopropane-1-carboxylic acid (ACC) is synthesized in the roots of plants and subsequently transported to the stem through the xylem, thus mediating plant flooding tolerance. Increased expression of ERFs and ethylene synthesis-related genes was observed in plants such as *Arabidopsis*, chrysanthemum, sesame and corn during waterlogging events (Wang et al., 2016; Yeung et al., 2018; Zhao et al., 2018; Dossa et al., 2019; Yu et al., 2019, 2020). Additionally, regulation of auxin signals also plays a crucial role by modulating germ elongation and development, secondary metabolism, carbohydrate metabolism, and mitochondrial electron transfer, under submerged conditions in rice (Wu and Yang, 2020). JA also participates in multiple aspects of the plant recovery phase. It primarily regulates beneficial rhizosphere bacteria-triggered induced systemic resistance, thereby playing a pivotal role in defense responses. *Pterocarya stenoptera* enhances waterlogging tolerance by increasing the synthesis of methyl linolenic acid and flavonoids, while activating JA signaling pathways (Li et al., 2021). In *A. thaliana* and rice, JA restricts root growth to minimize energy consumption under submergence conditions (Chen et al., 2019; Shukla et al., 2020). In *Arabidopsis*, ABA may also influence recovery from flooding stress through interaction with ethylene (Yeung et al., 2018).

## 3 The role of flooding-affected proteomics profile in plants

**Figure 1:**
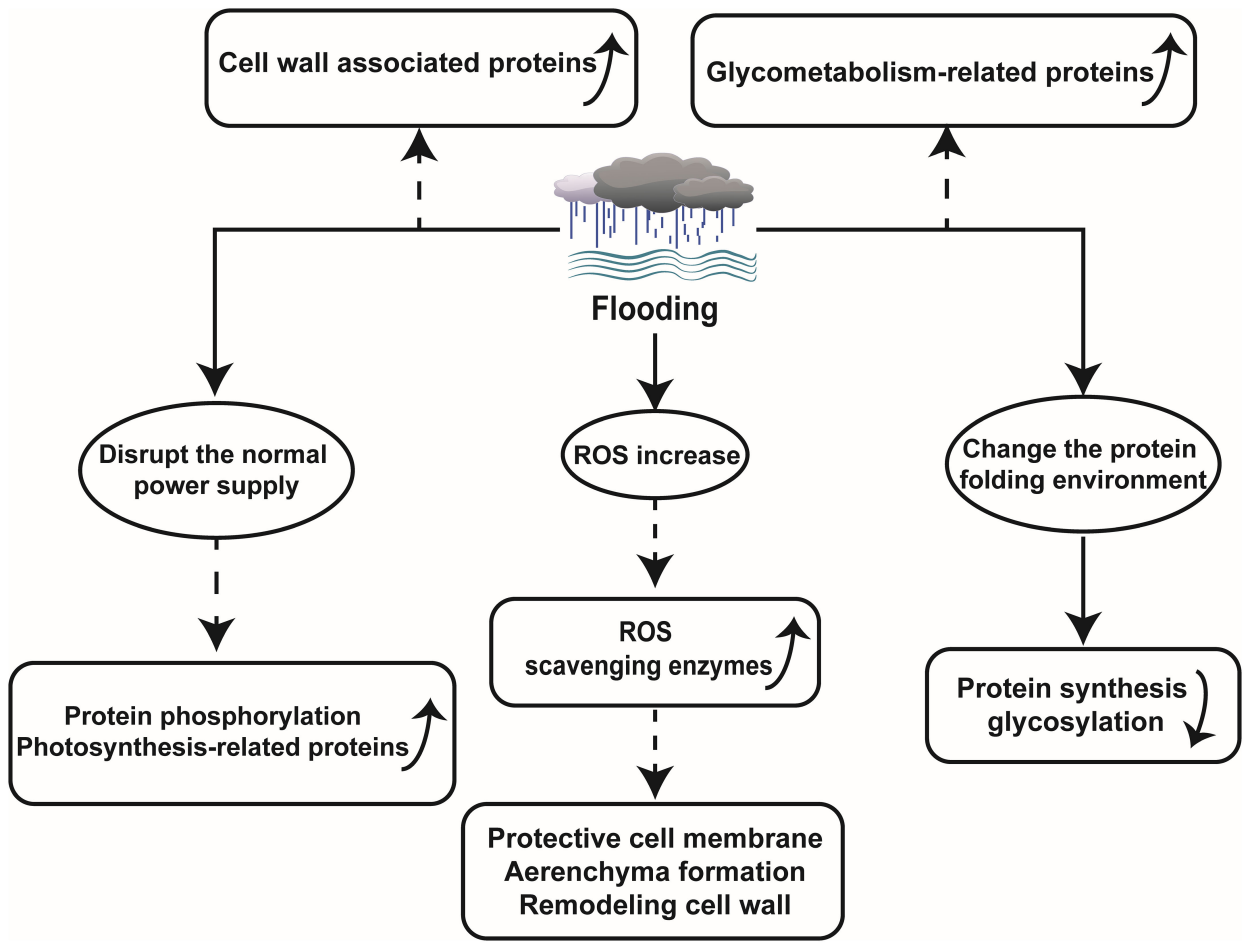
Schematic diagram of the effect of flooding on plant proteomics.

The proteomics technology encompasses quantifying protein abundance, expression levels, turnover rates, protein sequences, and post-translational modifications. Differential abundance detection and significant changes in physiological parameters serve as key methods in proteomics research. Proteins play a crucial role in plant responses to abiotic stresses such as flooding. When faced with environmental alterations, plants adjust their physiological traits to adapt. Accordingly, proteins directly participate in shaping new phenotypes (Rafique, 2019; Sharma et al., 2019). However, flooding tends to disrupt certain transcriptional and translational processes of proteins, which significantly impact protein abundance. For instance, during flooding stress on wheat seedlings, numerous alcohol dehydrogenase enzymes, fermentation-related enzymes, and other anaerobic adaptation-related proteins exhibit substantial increases, while most proteins involved in protein synthesis and targeting display significant decreases (Pan et al., 2019). Therefore, utilizing proteomic techniques to detect specific protein changes during flooding aids unraveling plant strategies for coping with or adapting to flooding stress, while elucidating fundamental principles underlying plant adaptations within flooding environments (**Figure 1**).

### 3.1 ROS levels increased and related proteins changed ROS levels increased and related proteins changed under flooding stress

Flooding can directly induce plant hypoxia, disrupting the energy balance within plant and perturbing the equilibrium of ROS production and scavenging. In rice, it was observed that under submerged conditions, electron transfer in mitochondria and chloroplasts was impaired, leading to inhibition of photosynthetic rate and reduction in cellular energy charge, consequently resulting in ROS accumulation. There was an upregulation of ROS scavenging protein (peroxidase), which led to a decrease in MDA content and O_2_- formation rate, while activating the antioxidant system to regulate ROS scavenging pathways in order to maintain the equilibrium of ROS at non-toxic level (Xiong et al., 2019). Peroxidase also plays a crucial role in safeguarding plant cell membranes, facilitating aerenchyma formation, and modulating cell wall remodeling in Maize (Hofmann et al., 2020). In the investigation on *Kandelia candel Druce*, a highly flooding resistant plant, flooding triggered an elevation in leaf ROS levels that phosphorylated serine/threonine protein kinases and activated MAPKs recognizing these phosphorylation sites (Pan et al., 2018). This mechanism is also present in *Arabidopsis thaliana*. Flooding leads to increased ROS levels prompting the activation of MAPK6 for enhanced survival under hypoxic conditions in *A. thaliana* (Chang et al., 2012).

### 3.2 Regulates pathway proteins related to glucose metabolism and protein phosphorylation under flooding

A stable supply of energy is indispensable for plants to withstand various stresses. Under flooding stress conditions, plants experience limited energy supply for their metabolic processes while having restricted soluble sugar content. Therefore, efficient sugar metabolism is crucial for plants to withstand damage caused by flooding stress. In Alfalfa, starch and sucrose metabolism related proteins were largely enriched during waterlogging stress (Zeng et al., 2019). It was reported that sucrose/starch metabolism and glycolysis were inhibited during flooding treatment of soybean seedlings, while plant-derived effectively alleviated the inhibition and enhances soybean growth during recovery from flooding stress. However, growth resumed due to the accumulation of amylase, glucan phosphorylase, and 4-glucan transferase proteins associated with starch and sucrose metabolism (Li et al., 2018b). In cotyledon of soybean, a total of 165 proteins were identified under flooding stress. Among them, 13 proteins were related to sucrose metabolism, indicating the importance of sucrose metabolism during plant responses to flooding stress (Kamal et al., 2015). Furthermore, millimeter wave irradiation on soybean seedlings activated sugar metabolism such as trehalose synthesis and positively regulated soybean growth under waterlogging stress by modulating glycolysis and REDOX-related pathway proteins (Zhong et al., 2020). Furthermore, barley subjected to waterlogging stress exhibited elevated expression levels of pyruvate decarboxylase (PDC), 1-amino cyclopropane 1-carboxylic acid oxidase (ACO), GST, as well as other proteins associated with photosynthesis, metabolism, and energy (Luan et al., 2018). Oxidative phosphorylation also occurs under flooding stress conditions. Yan et al. speculated that this ability may primarily originate from aleurone cells, as microscopic observation revealed a high abundance of mitochondria in these cells, which can provide energy for oxidative phosphorylation in the wheat seed endosperm (Yan et al., 2021). The study further demonstrated a significant down-regulation of proteins with peptidase activity or proteasome complexes under flooding stress. All these findings indicate that various plants employ distinct strategies to modulate their sugar metabolism to maintain energy homeostasis and adapt to the challenges posed by waterlogging.

Protein phosphorylation serves as a common post-translational regulatory mechanism enabling plants, such as *A. thaliana*, wheat, *K. candel*, and rapeseed, to acquire stable energy supply during flooding stress response (Mao et al., 2010; Chang et al., 2012; Pan et al., 2018; Xu et al., 2018).

### 3.3 Flooding causes changes in organelles, cell walls and other proteins

The accumulation of proteins associated with cell wall metabolism plays a crucial role in enhancing flooding tolerance in soybeans. Experimental evidence suggests that smoke treatment can enhance soybean growth by increasing the protein abundance and gene expression of O-fucosyltransferase family proteins associated with cell wall remodeling, thereby improving post-flooding survival rates (Li et al., 2018b). Similarly, melatonin supplementation has been shown to enrich proteins involved in cell wall metabolism and enhance plant resilience against flooding stress (Wang et al., 2021b). Under waterlogging conditions, the expression levels of Xyloglucan endotransglycosylase-1 (xet-1) and lignin biosynthesis-related proteins within the cell wall were significantly upregulated in ZS9 (tolerant) and GH01 (sensitive) rapeseed cultivars. This is because plants enhance cell adaptability through cell wall expansion and regulate the synthesis of polysaccharides and lignin to mitigate the impact of abiotic stress, thereby maintaining a balanced biological environment (Xu et al., 2018). Xylan is an indispensable polysaccharide in plant cell wall, and its synthesis is accomplished through xet-1 mediated reaction. In the face of extreme environmental conditions such as flooding, plants up-regulate the expression level of xet-1 gene, thereby promoting the biosynthesis process of xylan to enhance cell wall stability and stress resistance. Polysaccharides and lignin are pivotal constituents of the plant cell wall that not only fortify its stability but also regulate water permeability, preventing excessive expansion caused by over-absorption of water (Liu et al., 2018; Shu et al., 2021). Consequently, under abiotic stress conditions, plants augment polysaccharide and lignin biosynthesis to uphold cell wall integrity and maintain appropriate water permeability.

Moreover, recently developed proteomic techniques, subcellular proteomics, have yielded remarkable findings in elucidating the mechanisms underlying plant responses to flooding (Komatsu et al., 2012; Komatsu and Hashiguchi, 2018). As a powerful tool to elucidate localized cellular responses and subcellular communication, subcellular proteomics has vast potential to understand flooding-response mechanisms in plants. Using this technology, investigators found that flooding exerts a profound impact on the endoplasmic reticulum (ER), as it disrupts protein synthesis and glycosylation processes primarily by altering the protein folding environment and impeding calprotectin/calponin cycle-associated protein folding in soybean roots (Wang and Komatsu, 2016; Komatsu and Hashiguchi, 2018).

## 4 Metabolomics analysis of the effects of waterlogging on plant development

**Figure 2:**
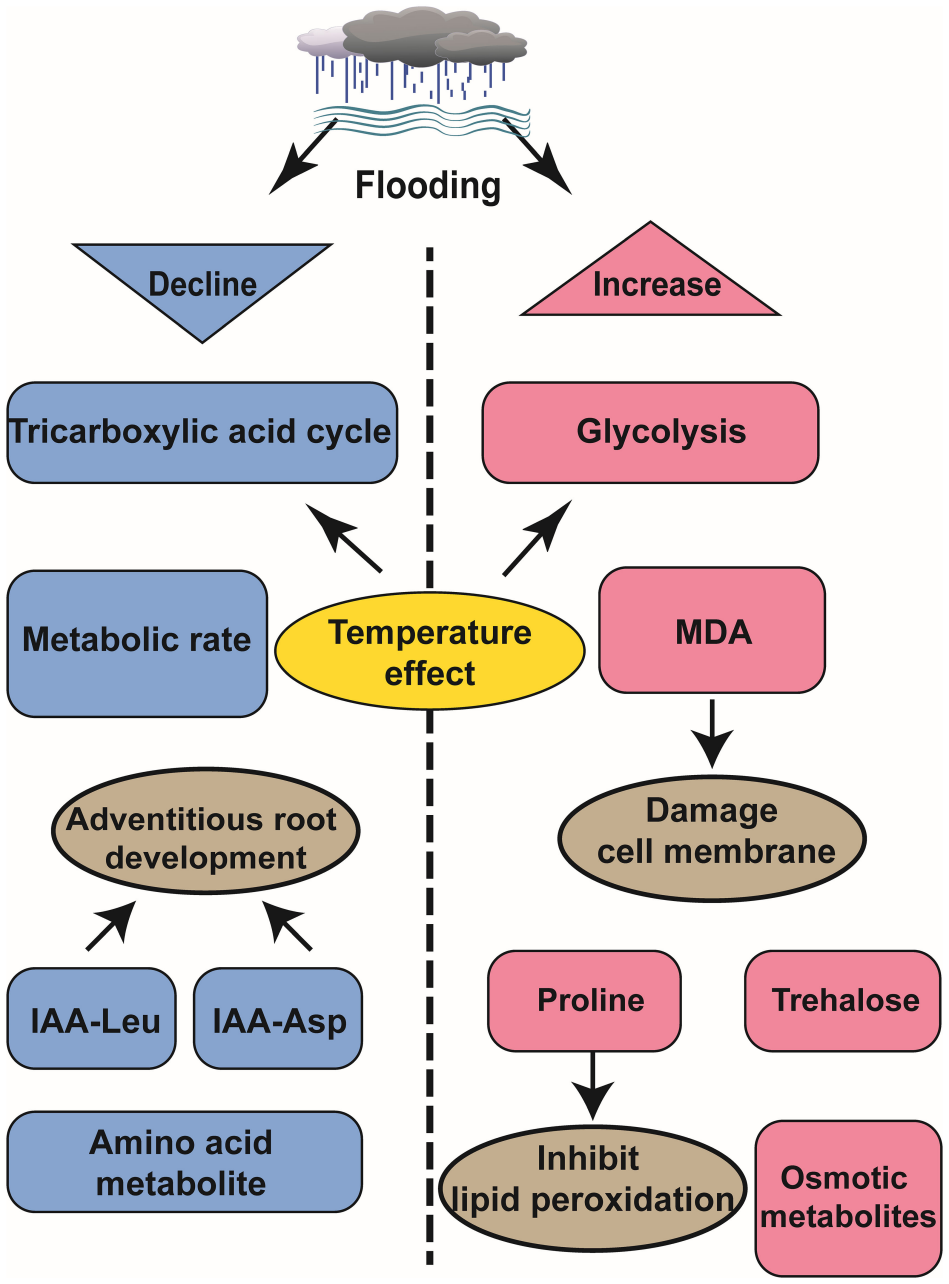
Schematic diagram of metabolomics analysis of the effects of waterlogging on plant development.

Metabolomics is an interdisciplinary and technologically integrated field that involves the separation and identification of biological metabolites at a specific time, data analysis and comparison, among other techniques. As fundamental components of the biological phenotype, metabolites serve as natural phenotypic indicators, enabling us to gain a more intuitive and effective understanding of biological processes and mechanisms (Zhang et al., 2021). Flooding trigger a comprehensive restructuring of plant primary metabolism to ensure cellular survival (Ruperti et al., 2019). Comparative analysis of metabolite accumulation levels offers valuable insights into the response mechanisms employed by plants during flooding events (**Figure 2**).

### 4.1 Plants provide energy by adjusting their metabolism under flooding

Flooding induces hypoxic conditions that impede respiration and metabolism, thereby disrupting the energy supply in plants (Hofmann et al., 2020). However, plants regulate sugar metabolism via the tricarboxylic acid (TCA) cycle and enhance the glycolysis pathway to sustain metabolic energy. In *Zostera marina*, this strategy is key to growing in the water and adapting to the oxygen-deprived environment. Studies have shown that the expression of glyceraldehyde 3-phosphate and related intermediates in glycolysis pathway is significantly upregulated under anoxic environment in water, thereby promoting anaerobic respiration through glycolysis (Zhang et al., 2021). In *Triticum aestivum*, both sugar and sugar phosphate are consumed in the germ and roots at temperatures ranging from 24°C to 28°C but not at temperatures between 15°C to 20°C. This indicates that temperature influences both the TCA cycle and sugar metabolism processes, contributing to variations in plant tolerance towards flooding stress (Huang et al., 2018). Flooding treated grape roots convert citric acid into succinic acid and adenosine triphosphate, while exhibiting a significant increase in alanine levels during flooding recovery that remains elevated for one week thereafter (Ruperti et al., 2019). Waterlogging-tolerant rice varieties employ escape strategies to cope with waterlogging stress. A study comparing deep-water variety C9285 with non-deep-water variety Taichung 65 (T65) revealed lower metabolic activity in C9285 under waterlogging conditions. The auxin signaling pathway plays a role in regulating secondary metabolism and carbohydrate metabolism while influencing adventitious root growth. Conjugants IAA-Leu and IAA-Asp exhibit reduced levels in C9285 compared to T65 over time (Fukushima et al., 2020).

### 4.2 Plant organs have different coping strategies resistance to flooding

Through metabolomic analysis of various plant organs, distinct response and adaptation mechanisms across different organs was observed under flooding events. Taking soybean as an example, under flooding stress, the alanine (Ala), gamma-aminobutyric acid (GABA), sucrose, acetic acid, citric acid, and succinic acid in leaves predominantly decrease; however, these substances show increased accumulation in soybean roots under the same environment (Coutinho et al., 2018). Flooding primarily induces anoxia and osmotic stress in root cells, leading to a reliance on glycolysis and fermentation for ATP production by the roots. Additionally, soybean roots accumulate trehalose (typically present at low levels in plants) to enhance survival under flooding conditions. In contrast to roots experiencing anoxic conditions during flooding stress, leaves do not encounter such oxygen deprivation (Coutinho et al., 2018).

Similarly, in the context of wheat response to flooding, anaerobic root fermentation serves as the primary mechanism for local root adaptation, while carbohydrate metabolism plays a pivotal role in orchestrating the overall systemic response. In addition, the highest content of alanine was found in the metabolite detection of xylem exudates, because alanine not only acts as a nitrogen source supplier, but also as a necessary carrier for carbon skeleton synthesis of glucose to meet the energy demand of wheat in waterlogging (Cid et al., 2023). Similar observations were reported by Jérémy Lothier et al., submerging *Medicago truncatula* root systems impeded sugar importation resulting in elevated phloem sugar reservoirs and reduced organic acid content; thus negatively regulated sugar metabolism in leaves and other aboveground tissues (Lothier et al., 2020).

### 4.3 Flooding leads to a decrease in amino acid metabolites

Amino acid metabolites, such as asparagine, arginine, and homoserine, decrease over time, whereas osmotic fluid-associated metabolites accumulate, like raffinose, galactinol, phenylpropanoids under flooding stress (Fukushima et al., 2020). Wheat tolerant varieties, like rice, employ escape strategies. Principal component analysis (PCA) of wheat cultivars Frument (intolerant) and Jackson (tolerant) revealed that flooding intolerant wheat varieties exhibited stronger metabolic reactions. Additionally, the results demonstrated that proline accumulation in tolerant varieties was a feedback response to flooding, aiding in the inhibition of lipid peroxidation during inundation. Notably, at the end of the submergence period, intolerant varieties experienced a significant increase in malondialdehyde (MDA) levels, leading to cell membrane damage (Herzog et al., 2018).

### 4.4 The enhanced influence of flooding on flavonoid synthesis

Although waterlogging has detrimental effects on plants, they can serve as a source of inspiration. The wild chrysanthemum, “Hangju”, is an authentic medicinal material containing flavonoids as its primary medicinal constituents. It has been observed that flooding-induced stress influences the expression of key enzymes involved in the synthesis pathway of flavonoids, thereby augmenting the accumulation of flavonoids and enhancing the efficacy of “Hangju” to a certain extent (Wang et al., 2019). Similarly, flooding exerts an influence on orchardgrass roots, thereby impacting crucial metabolic pathways such as flavonoid and amino acid metabolism (Shang et al., 2023).

## 5 Concluding remarks and future perspectives

In this paper, we provide a comprehensive review of staple crop flooding resistance strategies from the perspectives of metabolomics, proteomics, and transcriptomics. By integrating metabolomic and proteomic data, we were able to evaluate the impact of flooding on plant growth and development while elucidating changes in plant traits. Furthermore, transcriptomic analysis enables the construction of gene expression profiles associated with flooding events and facilitates the discovery of novel transcriptional regulatory mechanisms. Overall, reaching an equilibrium between energy production and consumption is of paramount importance for plants to withstand flooding.

At present, numerous challenges remain to be addressed, such as the mechanisms by which plants differentiate between short-term and long-term flooding stress and elicit distinct responses to each type of stress. In natural settings, flooding stress is often accompanied by low light stress, salt stress, temperature stress, etc. How do plants coordinate their responses and tolerate multiple concurrent stresses? How do different hormones coordinate plant growth and adaptation under flooding conditions? What are the common and specific genetic bases and molecular mechanisms underlying plant responses to flooding at various developmental stages? How do organelles respond to stress, and how does flooding impact crop yield and nutrient composition? It is believed that advancements in omics technology will lead to a more comprehensive understanding of the control network for plant flooding response, thereby providing a crucial theoretical foundation for agricultural production optimization of planting arrangements, improvement of crop resistance to flooding events, and ensuring food security.

However, the development of new omics techniques offers the possibility to answer these questions. Long-read sequencing platforms such as Oxford Nanopore Technologies (ONT) and PacBio have been widely used, providing opportunities for genome sequencing at the chromosome level in extremely flooding tolerance plants. Novel RNA sequencing technologies, such as Direct RNA Sequencing (DRS) and Iso-seq, help us discover more new transcripts and RNA modifications that are related to flooding. DNA-protein interaction technologies, such as ChIP-seq, CUT&Tag, Whole genome bisulfite sequencing (DAP-seq), etc., allow us to explore potential binding site information for specific flooding-tolerant transcription factors. DNA methylation research omics technologies such as Whole genome bisulfite sequencing (WGBS), Methyl-binding domain sequencing (MBD-seq) and Reduced-representation bisulfite sequencing (RRBS-seq), as well as histone modification research technologies such as ChIP-seq and CUT&Tag, have opened the door to a new world for us to study the epigenetic regulation mechanism of plant flooding tolerance. Single-cell omics and spatial transcriptomics techniques combine the expression abundance of mRNA with spatial location, providing important information on the relationship between cell function, phenotype, and location in the tissue microenvironment, which may provide a new aspect to our understanding of the mechanism of plant flooding tolerance. The development of these new omics techniques will certainly provide great opportunities for us to understand and elucidate the molecular mechanism of flooding tolerance in plants.

## References

[B1] Abdelrahman M. Burritt D. J. Tran L. P. (2018). The use of metabolomic quantitative trait locus mapping and osmotic adjustment traits for the improvement of crop yields under environmental stresses. Semin. Cell Dev. Biol. 83, 86–94. doi: 10.1016/j.semcdb.2017.06.020 28668354

[B2] Bailey-Serres J. Colmer T. D. (2014). Plant tolerance of flooding stress–recent advances. Plant Cell Environ. 37, 2211–2215. doi: 10.1111/pce.12420 25074340

[B3] Bailey-Serres J. Lee S. C. Brinton E. (2012). Waterproofing crops: effective flooding survival strategies. Plant Physiol. 160, 1698–1709. doi: 10.1104/pp.112.208173 23093359 PMC3510103

[B4] Bashar K. K. Tareq M. Z. Amin M. R. Honi U. Tahjib-Ul-Arif M. Sadat M. A.. (2019). Phytohormone-mediated stomatal response, escape and quiescence strategies in plants under flooding stress. Agronomy 9, 43. doi: 10.3390/agronomy9020043

[B5] Butsayawarapat P. Juntawong P. Khamsuk O. Somta P. (2019). Comparative transcriptome analysis of waterlogging-sensitive and tolerant zombi pea (Vigna vexillata) reveals energy conservation and root plasticity controlling waterlogging tolerance. Plants (Basel) 8, 264. doi: 10.3390/plants8080264 31382508 PMC6724125

[B6] Chang R. Jang C. J. H. Branco-Price C. Nghiem P. Bailey-Serres J. (2012). Transient MPK6 activation in response to oxygen deprivation and reoxygenation is mediated by mitochondria and aids seedling survival in Arabidopsis. Plant Mol. Biol. 78, 109–122. doi: 10.1007/s11103-011-9850-5 22086331

[B7] Chen X. Marszałkowska M. Reinhold-Hurek B. (2019). Jasmonic acid, not salicyclic acid restricts endophytic root colonization of rice. Front. Plant Sci. 10, 1758. doi: 10.3389/fpls.2019.01758 32063914 PMC7000620

[B8] Cid G. A. Francioli D. Kolb S. Tandron Moya Y. A. Von Wirén N. Hajirezaei M. R. (2023). Elucidating the systemic response of wheat plants under waterlogging based on transcriptomic and metabolic approaches. J. Exp. Bot. 75, 1510–1529. doi: 10.1093/jxb/erad453 38014629

[B9] Coutinho I. D. Henning L. M. M. Döpp S. A. Nepomuceno A. Moraes L. Marcolino-Gomes J.. (2018). Flooded soybean metabolomic analysis reveals important primary and secondary metabolites involved in the hypoxia stress response and tolerance. Environ. Exp. Bot. 153, 176–187. doi: 10.1016/j.envexpbot.2018.05.018

[B10] Dossa K. Mmadi M. A. Zhou R. Zhang T. Su R. Zhang Y.. (2019). Depicting the core transcriptome modulating multiple abiotic stresses responses in sesame (Sesamum indicum L.). Int. J. Mol. Sci. 20, 3930. doi: 10.3390/ijms20163930 31412539 PMC6721054

[B11] Dubois M. Van Den Broeck L. Inzé D. (2018). The pivotal role of ethylene in plant growth. Trends Plant Sci. 23, 311–323. doi: 10.1016/j.tplants.2018.01.003 29428350 PMC5890734

[B12] Fukushima A. Kuroha T. Nagai K. Hattori Y. Kobayashi M. Nishizawa T.. (2020). Metabolite and phytohormone profiling illustrates metabolic reprogramming as an escape strategy of deepwater rice during partially submerged stress. Metabolites 10, 68. doi: 10.3390/metabo10020068 32075002 PMC7074043

[B13] Herzog M. Fukao T. Winkel A. Konnerup D. Lamichhane S. Alpuerto J. B.. (2018). Physiology, gene expression, and metabolome of two wheat cultivars with contrasting submergence tolerance. Plant Cell Environ. 41, 1632–1644. doi: 10.1111/pce.13211 29664146

[B14] Hofmann A. Wienkoop S. Harder S. Bartlog F. Lüthje S. (2020). Hypoxia-responsive class III peroxidases in maize roots: soluble and membrane-bound isoenzymes. Int. J. Mol. Sci. 21, 8872. doi: 10.3390/ijms21228872 33238617 PMC7700428

[B15] Huang S. Shingaki-Wells R. N. Petereit J. Alexova R. Millar A. H. (2018). Temperature-dependent metabolic adaptation of Triticum aestivum seedlings to anoxia. Sci. Rep. 8, 6151. doi: 10.1038/s41598-018-24419-7 29670175 PMC5906562

[B16] Hwang J. H. Yu S. I. Lee B. H. Lee D. H. (2020). Modulation of energy metabolism is important for low-oxygen stress adaptation in brassicaceae species. Int. J. Mol. Sci. 21, 1787. doi: 10.3390/ijms21051787 32150906 PMC7084654

[B17] Jackson M. B. Colmer T. D. (2005). Response and adaptation by plants to flooding stress. Ann. Bot. 96, 501–505. doi: 10.1093/aob/mci205 16217870 PMC4247020

[B18] Kęska K. Szcześniak M. W. Makałowska I. Czernicka M. (2021). Long-term waterlogging as factor contributing to hypoxia stress tolerance enhancement in cucumber: comparative transcriptome analysis of waterlogging sensitive and tolerant accessions. Genes (Basel) 12, 189. doi: 10.3390/genes12020189 33525400 PMC7912563

[B19] Kamal A. H. M. Rashid H. Sakata K. Komatsu S. (2015). Gel-free quantitative proteomic approach to identify cotyledon proteins in soybean under flooding stress. J. Proteomics 112, 1–13. doi: 10.1016/j.jprot.2014.08.014 25201076

[B20] Kaur G. Vikal Y. Kaur L. Kalia A. Mittal A. Kaur D.. (2021). Elucidating the morpho-physiological adaptations and molecular responses under long-term waterlogging stress in maize through gene expression analysis. Plant Sci. 304, 110823. doi: 10.1016/j.plantsci.2021.110823 33568312

[B21] Klaas M. Haiminen N. Grant J. Cormican P. Finnan J. Arojju S. K.. (2019). Transcriptome characterization and differentially expressed genes under flooding and drought stress in the biomass grasses Phalaris arundinacea and Dactylis glomerata. Ann. Bot. 124, 717–730. doi: 10.1093/aob/mcz074 31241131 PMC6821378

[B22] Komatsu S. Hashiguchi A. (2018). Subcellular proteomics: application to elucidation of flooding-response mechanisms in soybean. Proteomes 6, 13. doi: 10.3390/proteomes6010013 29495455 PMC5874772

[B23] Komatsu S. Hiraga S. Yanagawa Y. (2012). Proteomics techniques for the development of flood tolerant crops. J. Proteome Res. 11, 68–78. doi: 10.1021/pr2008863 22029422

[B24] Komatsu S. Shirasaka N. Sakata K. (2013). ‘Omics’ techniques for identifying flooding-response mechanisms in soybean. J. Proteomics 93, 169–178. doi: 10.1016/j.jprot.2012.12.016 23313220

[B25] Li Z. Bai D. Zhong Y. Lin M. Sun L. Qi X.. (2022). Full-length transcriptome and RNA-seq analyses reveal the mechanisms underlying waterlogging tolerance in kiwifruit (Actinidia valvata). Int. J. Mol. Sci. 23, 3237. doi: 10.3390/ijms23063237 35328659 PMC8951935

[B26] Li J.-R. Liu C.-C. Sun C.-H. Chen Y.-T. (2018a). Plant stress RNA-seq Nexus: a stress-specific transcriptome database in plant cells. BMC Genomics 19, 966. doi: 10.1186/s12864-018-5367-5 30587128 PMC6307140

[B27] Li X. Rehman S. U. Yamaguchi H. Hitachi K. Tsuchida K. Yamaguchi T.. (2018b). Proteomic analysis of the effect of plant-derived smoke on soybean during recovery from flooding stress. J. Proteomics 181, 238–248. doi: 10.1016/j.jprot.2018.04.031 29704570

[B28] Li Y. Shi L. C. Yang J. Qian Z. H. He Y. X. Li M. W. (2021). Physiological and transcriptional changes provide insights into the effect of root waterlogging on the aboveground part of Pterocarya stenoptera. Genomics 113, 2583–2590. doi: 10.1016/j.ygeno.2021.06.005 34111522

[B29] Li C. Zhang B. (2016). MicroRNAs in control of plant development. J. Cell Physiol. 231, 303–313. doi: 10.1002/jcp.25125 26248304

[B30] Liu Z. Kumari S. Zhang L. Zheng Y. Ware D. (2012). Characterization of miRNAs in Response to Short-Term Waterlogging in Three Inbred Lines of Zea mays. PloS One 7, e39786. doi: 10.1371/journal.pone.0039786 22768123 PMC3387268

[B31] Liu Q. Luo L. Zheng L. (2018). Lignins: biosynthesis and biological functions in plants. Int. J. Mol. Sci. 19, 335. doi: 10.3390/ijms19020335 29364145 PMC5855557

[B32] Lothier J. Diab H. Cukier C. Limami A. M. Tcherkez G. (2020). Metabolic responses to waterlogging differ between roots and shoots and reflect phloem transport alteration in medicago truncatula. Plants (Basel) 9, 1373. doi: 10.3390/plants9101373 33076529 PMC7650564

[B33] Luan H. Shen H. Pan Y. Guo B. Lv C. Xu R. (2018). Elucidating the hypoxic stress response in barley (Hordeum vulgare L.) during waterlogging: A proteomics approach. Sci. Rep. 8, 9655. doi: 10.1038/s41598-018-27726-1 29941955 PMC6018542

[B34] Manghwar H. Hussain A. Alam I. Khoso M. A. Ali Q. Liu F. (2024). Waterlogging stress in plants: Unraveling the mechanisms and impacts on growth, development, and productivity. Environ. Exp. Bot. 224, 105824. doi: 10.1016/j.envexpbot.2024.105824

[B35] Mao X. Zhang H. Tian S. Chang X. Jing R. (2010). TaSnRK2.4, an SNF1-type serine/threonine protein kinase of wheat (Triticum aestivum L.), confers enhanced multistress tolerance in Arabidopsis. J. Exp. Bot. 61, 683–696. doi: 10.1093/jxb/erp331 20022921 PMC2814103

[B36] Nadarajah K. K. (2020). ROS homeostasis in abiotic stress tolerance in plants. Int. J. Mol. Sci. 21, 5208. doi: 10.3390/ijms21155208 32717820 PMC7432042

[B37] Pan R. He D. Xu L. Zhou M. Li C. Wu C.. (2019). Proteomic analysis reveals response of differential wheat (Triticum aestivum L.) genotypes to oxygen deficiency stress. BMC Genomics 20, 60. doi: 10.1186/s12864-018-5405-3 30658567 PMC6339445

[B38] Pan D. Wang L. Tan F. Lu S. Lv X. Zaynab M.. (2018). Phosphoproteomics unveils stable energy supply as key to flooding tolerance in Kandelia candel. J. Proteomics 176, 1–12. doi: 10.1016/j.jprot.2018.01.008 29353021

[B39] Pucciariello C. Perata P. (2021). The oxidative paradox in low oxygen stress in plants. Antioxid. (Basel) 10, 332. doi: 10.3390/antiox10020332 PMC7926446 33672303

[B40] Rafique S. (2019). Differential expression of leaf proteome of tolerant and susceptible maize (Zea mays L.) genotypes in response to multiple abiotic stresses. Biochem. Cell Biol. 97, 581–588. doi: 10.1139/bcb-2018-0338 30807207

[B41] Raza A. Su W. Gao A. Mehmood S. S. Hussain M. A. Nie W.. (2021). Catalase (CAT) gene family in rapeseed (Brassica napus L.): genome-wide analysis, identification, and expression pattern in response to multiple hormones and abiotic stress conditions. Int. J. Mol. Sci. 22, 4281. doi: 10.3390/ijms22084281 33924156 PMC8074368

[B42] Ruperti B. Botton A. Populin F. Eccher G. Brilli M. Quaggiotti S.. (2019). Flooding responses on grapevine: A physiological, transcriptional, and metabolic perspective. Front. Plant Sci. 10, 339. doi: 10.3389/fpls.2019.00339 30972087 PMC6443911

[B43] Sasidharan R. Bailey-Serres J. Ashikari M. Atwell B. J. Colmer T. D. Fagerstedt K.. (2017). Community recommendations on terminology and procedures used in flooding and low oxygen stress research. New Phytol. 214, 1403–1407. doi: 10.1111/nph.14519 28277605

[B44] Sasidharan R. Hartman S. Liu Z. Martopawiro S. Sajeev N. Van Veen H.. (2018). Signal dynamics and interactions during flooding stress. Plant Physiol. 176, 1106–1117. doi: 10.1104/pp.17.01232 29097391 PMC5813540

[B45] Shah T. Xu J. Zou X. Cheng Y. Nasir M. Zhang X. (2018). Omics approaches for engineering wheat production under abiotic stresses. Int. J. Mol. Sci. 19, 2390. doi: 10.3390/ijms19082390 30110906 PMC6121627

[B46] Shang P. Shen B. Zeng B. Bi L. Qu M. Zheng Y.. (2023). Integrated transcriptomic and metabolomics analysis of the root responses of orchardgrass to submergence stress. Int. J. Mol. Sci. 24, 2089. doi: 10.3390/ijms24032089 36768412 PMC9916531

[B47] Sharma J. K. Sihmar M. Santal A. R. Singh N. P. (2019). Impact assessment of major abiotic stresses on the proteome profiling of some important crop plants: a current update. Biotechnol. Genet. Eng. Rev. 35, 126–160. doi: 10.1080/02648725.2019.1657682 31478455

[B48] Sharma R. Upadhyay S. Bhat B. Singh G. Bhattacharya S. Singh A. (2020). Abiotic stress induced miRNA-TF-gene regulatory network: A structural perspective. Genomics 112, 412–422. doi: 10.1016/j.ygeno.2019.03.004 30876925

[B49] Shu F. Jiang B. Yuan Y. Li M. Wu W. Jin Y.. (2021). Biological activities and emerging roles of lignin and lignin-based products─A review. Biomacromolecules 22, 4905–4918. doi: 10.1021/acs.biomac.1c00805 34806363

[B50] Shukla V. Lombardi L. Pencik A. Novak O. Weits D. A. Loreti E.. (2020). Jasmonate signaling contributes to primary root inhibition upon oxygen deficiency in arabidopsis thaliana. Plants (Basel) 9, 1046. doi: 10.3390/plants9081046 32824502 PMC7464498

[B51] Voesenek L. A. Bailey-Serres J. (2013). Flooding tolerance: O2 sensing and survival strategies. Curr. Opin. Plant Biol. 16, 647–653. doi: 10.1016/j.pbi.2013.06.008 23830867

[B52] Wang X. He Y. Zhang C. Tian Y. A. Lei X. Li D.. (2021a). Physiological and transcriptional responses of Phalaris arundinacea under waterlogging conditions. J. Plant Physiol. 261, 153428. doi: 10.1016/j.jplph.2021.153428 33957505

[B53] Wang X. Komatsu S. (2016). Gel-free/label-free proteomic analysis of endoplasmic reticulum proteins in soybean root tips under flooding and drought stresses. J. Proteome Res. 15, 2211–2227. doi: 10.1021/acs.jproteome.6b00190 27224218

[B54] Wang X. Li F. Chen Z. Yang B. Komatsu S. Zhou S. (2021b). Proteomic analysis reveals the effects of melatonin on soybean root tips under flooding stress. J. Proteomics 232, 104064. doi: 10.1016/j.jprot.2020.104064 33276190

[B55] Wang L. Li D. Zhang Y. Gao Y. Yu J. Wei X.. (2016). Tolerant and susceptible sesame genotypes reveal waterlogging stress response patterns. PloS One 11, e0149912. doi: 10.1371/journal.pone.0149912 26934874 PMC4774966

[B56] Wang P. Yamaji N. Inoue K. Mochida K. Ma J. F. (2020). Plastic transport systems of rice for mineral elements in response to diverse soil environmental changes. New Phytol. 226, 156–169. doi: 10.1111/nph.16335 31758804

[B57] Wang Y. Zhang Y. Zhou R. Dossa K. Yu J. Li D.. (2018). Identification and characterization of the bZIP transcription factor family and its expression in response to abiotic stresses in sesame. PloS One 13, e0200850. doi: 10.1371/journal.pone.0200850 30011333 PMC6047817

[B58] Wang T. Zou Q. Guo Q. Yang F. Wu L. Zhang W. (2019). Widely targeted metabolomics analysis reveals the effect of flooding stress on the synthesis of flavonoids in chrysanthemum morifolium. Molecules 24, 3695. doi: 10.3390/molecules24203695 31615126 PMC6832227

[B59] Wu J. Wang J. Hui W. Zhao F. Wang P. Su C.. (2022). Physiology of plant responses to water stress and related genes: A review. Forests 13, 324. doi: 10.3390/f13020324

[B60] Wu Y. S. Yang C. Y. (2020). Comprehensive transcriptomic analysis of auxin responses in submerged rice coleoptile growth. Int. J. Mol. Sci. 21, 1292. doi: 10.3390/ijms21041292 32075118 PMC7072898

[B61] Xiong Q. Cao C. Shen T. Zhong L. He H. Chen X. (2019). Comprehensive metabolomic and proteomic analysis in biochemical metabolic pathways of rice spikes under drought and submergence stress. Biochim. Biophys. Acta Proteins Proteom. 1867, 237–247. doi: 10.1016/j.bbapap.2019.01.001 30611782

[B62] Xu J. Qiao X. Tian Z. Zhang X. Zou X. Cheng Y.. (2018). Proteomic analysis of rapeseed root response to waterlogging stress. Plants (Basel) 7, 71. doi: 10.3390/plants7030071 30205432 PMC6160990

[B63] Xu Z. Ye L. Shen Q. Zhang G. (2023). Advances in studies on waterlogging tolerance in plants. J. Integr. Agricult. doi: 10.1016/j.jia.2023.12.028

[B64] Yan M. Zheng L. Li B. Shen R. Lan P. (2021). Comparative proteomics reveals new insights into the endosperm responses to drought, salinity and submergence in germinating wheat seeds. Plant Mol. Biol. 105, 287–302. doi: 10.1007/s11103-020-01087-8 33104943

[B65] Yeung E. Van Veen H. Vashisht D. Sobral Paiva A. L. Hummel M. Rankenberg T.. (2018). A stress recovery signaling network for enhanced flooding tolerance in Arabidopsis thaliana. Proc. Natl. Acad. Sci. U.S.A. 115, E6085–e6094. doi: 10.1073/pnas.1803841115 29891679 PMC6042063

[B66] Yu F. Liang K. Fang T. Zhao H. Han X. Cai M.. (2019). A group VII ethylene response factor gene, ZmEREB180, coordinates waterlogging tolerance in maize seedlings. Plant Biotechnol. J. 17, 2286–2298. doi: 10.1111/pbi.13140 31033158 PMC6835127

[B67] Yu F. Tan Z. Fang T. Tang K. Liang K. Qiu F. (2020). A comprehensive transcriptomics analysis reveals long non-coding RNA to be involved in the key metabolic pathway in response to waterlogging stress in maize. Genes (Basel) 11, 267. doi: 10.3390/genes11030267 32121334 PMC7140884

[B68] Yuan L. B. Dai Y. S. Xie L. J. Yu L. J. Zhou Y. Lai Y. X.. (2017). Jasmonate regulates plant responses to postsubmergence reoxygenation through transcriptional activation of antioxidant synthesis. Plant Physiol. 173, 1864–1880. doi: 10.1104/pp.16.01803 28082717 PMC5338657

[B69] Zaman M. S. U. Malik A. I. Erskine W. Kaur P. (2019). Changes in gene expression during germination reveal pea genotypes with either “quiescence” or “escape” mechanisms of waterlogging tolerance. Plant Cell Environ. 42, 245–258. doi: 10.1111/pce.13338 29761495

[B70] Zeng N. Yang Z. Zhang Z. Hu L. Chen L. (2019). Comparative transcriptome combined with proteome analyses revealed key factors involved in alfalfa (Medicago sativa) response to waterlogging stress. Int. J. Mol. Sci. 20, 1359. doi: 10.3390/ijms20061359 30889856 PMC6471898

[B71] Zhang Y. Zhao P. Yue S. Liu M. Qiao Y. Xu S.. (2021). New insights into physiological effects of anoxia under darkness on the iconic seagrass Zostera marina based on a combined analysis of transcriptomics and metabolomics. Sci. Total Environ. 768, 144717. doi: 10.1016/j.scitotenv.2020.144717 33736305

[B72] Zhao N. Li C. Yan Y. Cao W. Song A. Wang H.. (2018). Comparative Transcriptome Analysis of Waterlogging-Sensitive and Waterlogging-Tolerant Chrysanthemum morifolium Cultivars under Waterlogging Stress and Reoxygenation Conditions. Int. J. Mol. Sci. 19, 1455. doi: 10.3390/ijms19051455 29757964 PMC5983694

[B73] Zhong Z. Furuya T. Ueno K. Yamaguchi H. Hitachi K. Tsuchida K.. (2020). Proteomic analysis of irradiation with millimeter waves on soybean growth under flooding conditions. Int. J. Mol. Sci. 21, 486. doi: 10.3390/ijms21020486 31940953 PMC7013696

[B74] Zhou W. Chen F. Meng Y. Chandrasekaran U. Luo X. Yang W.. (2020). Plant waterlogging/flooding stress responses: From seed germination to maturation. Plant Physiol. Biochem. 148, 228–236. doi: 10.1016/j.plaphy.2020.01.020 31981875

[B75] Zou X. Jiang Y. Liu L. Zhang Z. Zheng Y. (2010). Identification of transcriptome induced in roots of maize seedlings at the late stage of waterlogging. BMC Plant Biol. 10, 189. doi: 10.1186/1471-2229-10-189 20738849 PMC2956539

