# Supplementary material for: Application of multiomics analysis to plant flooding response
Source: Front Plant Sci. 2024 Aug 13;15:1389379. doi: 10.3389/fpls.2024.1389379 (PMC11347887; doi:10.3389/fpls.2024.1389379)
Supplement: Supplementary file 1 [file Table1.docx]

Application of Multiomics Analysis to Plant Flooding Response

Guangya Gui ^1,2†^, Qi Zhang ^1†^, Weiming Hu ^2*^ and Fen Liu ^2*^

^1^College of Traditional Chinese Medicine, Jiangxi University of Chinese Medicine, Nanchang, 330004, China.

^2^Lushan Botanical Garden, Jiangxi Province and Chinese Academy of Sciences, Jiujiang 332000, China.

^†^These authors contributed equally to this work.

*** Correspondence:**Fen Liu and Weiming Hu
liuf@lsbg.cn (F.L.); huwm@lsbg.cn (W.H.)

**Supplemental table 1.** A summary of omics studies on plant flooding response.

| Study | Species | Tissue type | Plant age | Stress treatment type | duration | Platform | Key points of interest |
| --- | --- | --- | --- | --- | --- | --- | --- |
| Transcriptomics |  |  |  |  |  |  |  |
| (Zeng et al., 2020) | *Dactylis glomerata* | Leaves | 6 months | Submergence | 3 days and 7 days | RNA-seq | Genes associated with REDOX system, nucleic acid-binding transcription factor activity and glycerol kinase activity are involved in regulation. |
| (Wu and Yang, 2020) | Rice | Seed | Seed | Submergence | 5 days | RNA-seq | Auxin signal transduction is involved in the regulation of secondary metabolism carbohydrate metabolism and mitochondrial electron transport under submerged conditions. |
| (Shang et al., 2023) | Orchardgrass | Root | When the plant has three to four leaves | Submergence | 0, 8, and 24 hours | RNA-seq | The main metabolic pathways of orchardgrass roots under inundation stress were identified. |
| (Yeung et al., 2018) | *Arabidopsis*  Bay-0, Lp2-6, Col-0, *rbohD-3* | Leaves | 10-leaf-stage plants | Submergence | 5 days | Ribo-Seq | A regulatory network that controls water loss and senescence to influence recovery was identified in *A. thaliana* under flooding. |
| (Yu et al., 2019) | Maize | Root | Seedling stage | Waterlogging | 4 hours and 3 days | RNA-seq | One gene, ZmEREB180, was strongly associated with waterlogging tolerance. |
| (Zeng et al., 2019) | Alfalfa | Whole plant | 5 weeks | Waterlogging | 12 days | RNA-seq | 57 flood-sensitive genes and related pathways were identified. |
| (Klaas et al., 2019) | *Dactylis glomerata*; *Phalaris arundinacea* | Leaves | 8 weeks | Waterlogging | 2 weeks | RNA-seq | Found several candidate genes involved in detoxification and degradation during flood. |
| (Wang et al., 2021a) | *Phalaris arundinacea* | Leaf and root | 2 weeks (Tillers) | Waterlogging | 6 weeks | RNA-seq | Genes related to metabolic process, cell, ribosome and phenyl-C biosynthesis pathway are involved in the regulation of plant flood stress. |
| (Zaman et al., 2019) | Pea | Seed | Seed | Waterlogging | 12 hours, 24 hours, 48 hours, 96 hours | RNA-seq | The related genes adapted to waterlogging stress during pea germination were identified. |
| (Wang et al., 2016) | Sesame | Root | Not mentioned | Waterlogging | 0, 3, 9, and 15 h under waterlogging stress as well as 20 h post-drainage | RNA-seq | 66 genes were identified as candidate genes for improving sesame tolerance to waterlogging. |
| (Dossa et al., 2019) | Sesame | Root | Not mentioned | Waterlogging | 3 hours | RNA-seq | Core abiotic stress response genes in sesame are invistigated from a meta-analysis of 72 RNA-Seq datasets from drought (Dossa et al., 2017), waterlogging (Wang et al., 2016), salt (Zhang et al., 2019) and osmotic stresses (this study). |
| (Zhao et al., 2018) | *Chrysanthemum morifolium* | Root | When the plant has 10-12 leaves | Waterlogging | 0 h, 12 h, and 12 h + 2 h (2 h reoxygenation) | RNA-seq | Ethylene, transcription factors and genes involved in hormone response, n-terminal regulation pathway and ROS signaling pathway are involved in flood regulation. |
| (Ruperti et al., 2019) | Grapevine | Root | 3 years | Waterlogging | 1, 2, 8, 16, and 21 days | RNA-seq | Summarized in waterlogged during and after the depth of the metabolism and transcriptional reprogramming. |
| (Li et al., 2021) | *Pterocarya stenoptera* | Leaves | 6 months | Waterlogging | 3, 6, 12, 24, 48 and 72 hours | RNA-seq | Activation of JA, ethylene and auxin signaling pathways to enhance waterlogging tolerance. |
| (Qi et al., 2020) | Cucumber | Hypocotyl | 3 weeks | Waterlogging | 7 days | RNA-seq | The emergence of adventitious roots is regulated by the interaction of sugars and auxin, but elongation is dependent only on sugars. |
| (Zou et al., 2010) | Maize | Root | Plants initiated three leaves in total with two leaves expanded. | Waterlogging | 12, 16, 20, and 24 hours | Suppression subtractive hybridization (SSH) and reverse northern blotting | Amino acid metabolism plays two main roles at the late stage of waterlogging: the maintance of cytoplasmic pH and energy supply through breakdown of the carbon skeleton. |
| (Liu et al., 2012) | Maize | Root | 2 weeks | Waterlogging | 1, 2, and 4 hours | small RNA-seq | A set of miRNA involved in waterlogging were identified. |
| (Yu et al., 2020) | Maize | Root tip | Second leaf stage | Waterlogging | Mixed sample (2, 4, 6, 8, 10, and 12 hours) | RNA-seq | Long non-coding RNA is involved in maize response to waterlogging stress. |
| (Kęska et al., 2021) | Cucumber | Root | 21 days | Waterlogging | 7 days waterlogging, 7 days of waterlogging and 14 days of recovery, 7 days waterlogging, and 14 days of recovery, then waterlogged 7 days again. | RNA-seq | The expression of genes related to glycolysis, advential root development and amino acid metabolism was significant for the long-term waterlogging tolerance of cucumber. |
| (Cid et al., 2023) | Wheat | Root and leaves | After the third leaf appears | Waterlogging | 12 days | RNA-seq | Alanine works as a vehicle for carbon skeletons to produce glucose *de novo* and meet the energy demand during waterlogging. |
| (Li et al., 2022) | Kiwifruit | Root | Five- to six-leaf stage (asexually propagated plants) | Waterlogging | 0, 12, 24, and 72 hours | RNA-seq  Iso-seq | A set of candidant genes reponse to waterlogging were identified in Kiwifruit. |
| (Hwang et al., 2020) | Brassicaceae Species (*Arabidopsis* *thaliana*, *Arabis stelleri*, *Rorippa islandica*, and *Thellungiella salsuginea*) | Root | 2 weeks (Arabidopsis)  3 weeks (Other species) | Hypoxia (Low-oxygen) treatment (0.1% O_2_/99.9% N_2_) | 0, 1, 3, 8, 24, or 72 hours | Microarray | Energy metabolic pathways of Brassicaceae Species under hypoxic stress indicate that plants developed different energy metabolisms to cope with the energy crisis caused by low-oxygen stress. |
| (Hofmann et al., 2020) | Maize | Root | 14 days | Hypoxia | 24 hours | RNA-seq | Peroxidase plays a role in membrane protection aerenchyma formation and cell wall remodeling under hypoxia conditions |
| (Zhang et al., 2021) | *Zostera marina* | Whole plant | 30 cm in length and more than five stem nodes | Anoxic treatment (<1% oxygen saturation in seawater) | 72 hours | RNA-seq | *Zostera marina* resists hypoxic stress by promoting anaerobic respiration and limiting aerobic respiration, as well as regulating nitrogen metabolism. |
| Proteomics |  |  |  |  |  |  |  |
| (Xiong et al., 2019) | Rice | Rice spikes | 40 days | Submergence | 1 days | LC-MS/MS | Submergence promoted the energy metabolism pathway, carbon fixation in photosynthetic organism pathway, carbohydrate metabolic process and reactive oxygen species metabolic process in rice. |
| (Pan et al., 2018) | *Kandelia candel* | Leaves | 40 days | Submergence | 6 hours | Nano LC-MS/MS | Protein phosphorylation is important in flood tolerance. |
| (Yan et al., 2020) | Wheat | Seed | 1/3 embryo-proximal seed parts | Submergence | 1 days | Nano LC-MS/MS | Flood affected the metabolism of cysteine and methionine as well as starch and sucrose. |
| (Wang and Komatsu, 2016) | Soybean | Root tip | 2 days | Submergence | 2 days | Endoplasmic Reticulum Enrichment/LC–MS/MS | 255, 368, 103 proteins in control, flooding, drought. |
| (Kamal et al., 2015) | Soybean | Cotyledon | 2 days | Submergence | 4 days | nano-LC–MS/MS | 165 proteins from cotyledon were identified under flooding stress. |
| (Wang et al., 2021b) | Soybean | Root tip | 2 days | Waterlogging | 2 days | HPLC-MS | Explain why melatonin enhances plant waterlogging tolerance. |
| (Zhong et al., 2020) | Soybean | Root-hypocotyl | 2 days and 4 days | Waterlogging | 2 days | Nano LC-MS/MS | Millimeter wave irradiation of soybean seeds can regulate glycolysis and REDOX related pathways to promote growth under waterlogging. |
| (Luan et al., 2018) | Barley | Leaves, adventitious roots, seminal roots and nodal roots | Four-leaf stage | Waterlogging | 3 weeks | 2D gel/MS | The biomass and photosynthetic performance of waterlogging intolerant varieties decreased more. |
| (Xu et al., 2018) | Rapeseed | Root | 36 hours (root or radicle grow to 5mm long) | Waterlogging | 0, 4, 8, and12 hours | LC-MS/MS | Rapeseed responded to floods in various ways. |
| (Li et al., 2018) | Soybean | Root and hypocotyl | 2 days | Waterlogging | 2 days | LC-MS/MS | Plant-derived smoke affects the balance of glucose metabolism and glycolysis and promotes the recovery of soybean under flooding stress. |
| (Zeng et al., 2019) | Alfalfa | Whole plant | 5 weeks | Waterlogging | 12 days | LC-MS/MS | A total of 187 (122 up- and 65 down-regulated) and 190 (105 up- and 85 down-regulated) DAPs were identified. |
| (Pan et al., 2019) | Wheat | Root | 12 days | Hypoxic treatment (hydroponics, oxygen content of water was controlled at 2.0 mg/L) | 0, 1, 2, and 3 days | LC-MS/MS | Proteins involved in primary metabolism and protein processing are vulnerable to flooding. |
| (Hofmann et al., 2020) | Maize | Root | 14 days | Hypoxia | 24 hours | 2D gel/MS | Peroxidase plays a role in membrane protection aerenchyma formation and cell wall remodeling under hypoxia conditions |
| Metabolomics |  |  |  |  |  |  |  |
| (Herzog et al., 2018) | Wheat | Whole plant | 18 days | Submergence | 0, 2, 5, 8, 12, 14 and 16 days | GC-MS; LC-MS | Relationship between water tolerance and carbohydrate consumption in shoot of two kinds of wheat. |
| (Shang et al., 2023) | Orchardgrass | Root | When the plant has three to four leaves | Submergence | 0, 8, and 24 hours | LC-MS | The main metabolic pathways of orchardgrass roots under inundation stress were identified. |
| (Xiong et al., 2019) | Rice | Rice spikes | 40 days | Submergence | 1 days | LC-MS | Submergence promoted the energy metabolism pathway, carbon fixation in photosynthetic organism pathway, carbohydrate metabolic process and reactive oxygen species metabolic process in rice. |
| (Fukushima et al., 2020) | Deepwater Rice | Leaves | 9–10 leaf stage | Partial submergence | 24, and 288 hours | GC-MS; CE-MS; UHPLC-ESI-qMS/MS | Metabolic reprogramming in deepwater rice is a plant escape strategy. |
| (Lothier et al., 2020) | *Medicago truncatula* | Roots and shoots | 3 weeks | Waterlogging | 7 days and 21 days | GC-MS | Immersion root inhibition sugar import. |
| (Wang et al., 2019) | *Chrysanthemum morifolium* | Flower bud | Not mentioned | Waterlogging | 3 days | LC-MS | Flood affects the expression of enzymes to change the accumulation of flavonoids. |
| (Cid et al., 2023) | Wheat | Root and leaf | After the third leaf appears | Waterlogging | 12 days | LC-MS | Whole-body response of wheat plants to waterlogging. |
| (Ruperti et al., 2019) | Grapevine | Root | 3 years | Waterlogging | 1, 2, 8, 16, and 21 days | Nuclear Magnetic Resonance Spectroscopy (NMR) | Summarized in waterlogged during and after the depth of the metabolism and transcriptional reprogramming. |
| (Huang et al., 2018) | Wheat | The roots and coleoptile | 4 days | Hypoxia | 1 day | GC-MS | Temperature affects waterlogging tolerance. |
| (Zhang et al., 2021) | *Zostera marina* | Whole plant | 30 cm in length and more than five stem nodes | Anoxic treatment (<1% oxygen saturation in seawater) | 72 hours | LC-MS | *Zostera marina* resists hypoxic stress by promoting anaerobic respiration and limiting aerobic respiration, as well as regulating nitrogen metabolism |
| (Coutinho et al., 2018) | Soybean | The third trifoliate leaf and root | V3 stage | Waterlogging | 3, 7 and 12 Days of Flooding | ^1^H 1D NMR | Flooding stress strongly affects both the primary and secondary soybean metabolome. |

**References:**

Cid, G.A., Francioli, D., Kolb, S., Tandron Moya, Y.A., Von Wirén, N., and Hajirezaei, M.R. (2023). Elucidating the systemic response of wheat plants under waterlogging based on transcriptomic and metabolic approaches. *J Exp Bot*.

Coutinho, I.D., Henning, L.M.M., Döpp, S.A., Nepomuceno, A., Moraes, L.a.C., Marcolino-Gomes, J., Richter, C., Schwalbe, H., and Colnago, L.A. (2018). Flooded soybean metabolomic analysis reveals important primary and secondary metabolites involved in the hypoxia stress response and tolerance. *Environmental and Experimental Botany* 153**,** 176-187.

Dossa, K., Li, D., Wang, L., Zheng, X., Liu, A., Yu, J., Wei, X., Zhou, R., Fonceka, D., Diouf, D., Liao, B., Cissé, N., and Zhang, X. (2017). Transcriptomic, biochemical and physio-anatomical investigations shed more light on responses to drought stress in two contrasting sesame genotypes. *Scientific Reports* 7**,** 8755.

Dossa, K., Mmadi, M.A., Zhou, R., Zhang, T., Su, R., Zhang, Y., Wang, L., You, J., and Zhang, X. (2019). Depicting the Core Transcriptome Modulating Multiple Abiotic Stresses Responses in Sesame (Sesamum indicum L.). *Int J Mol Sci* 20.

Fukushima, A., Kuroha, T., Nagai, K., Hattori, Y., Kobayashi, M., Nishizawa, T., Kojima, M., Utsumi, Y., Oikawa, A., Seki, M., Sakakibara, H., Saito, K., Ashikari, M., and Kusano, M. (2020). Metabolite and Phytohormone Profiling Illustrates Metabolic Reprogramming as an Escape Strategy of Deepwater Rice during Partially Submerged Stress. *Metabolites* 10.

Herzog, M., Fukao, T., Winkel, A., Konnerup, D., Lamichhane, S., Alpuerto, J.B., Hasler-Sheetal, H., and Pedersen, O. (2018). Physiology, gene expression, and metabolome of two wheat cultivars with contrasting submergence tolerance. *Plant Cell Environ* 41**,** 1632-1644.

Hofmann, A., Wienkoop, S., Harder, S., Bartlog, F., and Lüthje, S. (2020). Hypoxia-Responsive Class III Peroxidases in Maize Roots: Soluble and Membrane-Bound Isoenzymes. *Int J Mol Sci* 21.

Huang, S., Shingaki-Wells, R.N., Petereit, J., Alexova, R., and Millar, A.H. (2018). Temperature-dependent metabolic adaptation of Triticum aestivum seedlings to anoxia. *Sci Rep* 8**,** 6151.

Hwang, J.H., Yu, S.I., Lee, B.H., and Lee, D.H. (2020). Modulation of Energy Metabolism Is Important for Low-Oxygen Stress Adaptation in Brassicaceae Species. *Int J Mol Sci* 21.

Kamal, A.H.M., Rashid, H., Sakata, K., and Komatsu, S. (2015). Gel-free quantitative proteomic approach to identify cotyledon proteins in soybean under flooding stress. *Journal of Proteomics* 112**,** 1-13.

Kęska, K., Szcześniak, M.W., Makałowska, I., and Czernicka, M. (2021). Long-Term Waterlogging as Factor Contributing to Hypoxia Stress Tolerance Enhancement in Cucumber: Comparative Transcriptome Analysis of Waterlogging Sensitive and Tolerant Accessions. *Genes (Basel)* 12.

Klaas, M., Haiminen, N., Grant, J., Cormican, P., Finnan, J., Arojju, S.K., Utro, F., Vellani, T., Parida, L., and Barth, S. (2019). Transcriptome characterization and differentially expressed genes under flooding and drought stress in the biomass grasses Phalaris arundinacea and Dactylis glomerata. *Ann Bot* 124**,** 717-730.

Li, X., Rehman, S.U., Yamaguchi, H., Hitachi, K., Tsuchida, K., Yamaguchi, T., Sunohara, Y., Matsumoto, H., and Komatsu, S. (2018). Proteomic analysis of the effect of plant-derived smoke on soybean during recovery from flooding stress. *J Proteomics* 181**,** 238-248.

Li, Y., Shi, L.C., Yang, J., Qian, Z.H., He, Y.X., and Li, M.W. (2021). Physiological and transcriptional changes provide insights into the effect of root waterlogging on the aboveground part of Pterocarya stenoptera. *Genomics* 113**,** 2583-2590.

Li, Z., Bai, D., Zhong, Y., Lin, M., Sun, L., Qi, X., Hu, C., and Fang, J. (2022). Full-Length Transcriptome and RNA-Seq Analyses Reveal the Mechanisms Underlying Waterlogging Tolerance in Kiwifruit (Actinidia valvata). *International Journal of Molecular Sciences* 23**,** 3237.

Liu, Z., Kumari, S., Zhang, L., Zheng, Y., and Ware, D. (2012). Characterization of miRNAs in Response to Short-Term Waterlogging in Three Inbred Lines of Zea mays. *PLOS ONE* 7**,** e39786.

Lothier, J., Diab, H., Cukier, C., Limami, A.M., and Tcherkez, G. (2020). Metabolic Responses to Waterlogging Differ between Roots and Shoots and Reflect Phloem Transport Alteration in Medicago truncatula. *Plants (Basel)* 9.

Luan, H., Shen, H., Pan, Y., Guo, B., Lv, C., and Xu, R. (2018). Elucidating the hypoxic stress response in barley (Hordeum vulgare L.) during waterlogging: A proteomics approach. *Sci Rep* 8**,** 9655.

Pan, D., Wang, L., Tan, F., Lu, S., Lv, X., Zaynab, M., Cheng, C.L., Abubakar, Y.S., Chen, S., and Chen, W. (2018). Phosphoproteomics unveils stable energy supply as key to flooding tolerance in Kandelia candel. *J Proteomics* 176**,** 1-12.

Pan, R., He, D., Xu, L., Zhou, M., Li, C., Wu, C., Xu, Y., and Zhang, W. (2019). Proteomic analysis reveals response of differential wheat (Triticum aestivum L.) genotypes to oxygen deficiency stress. *BMC Genomics* 20**,** 60.

Qi, X., Li, Q., Shen, J., Qian, C., Xu, X., Xu, Q., and Chen, X. (2020). Sugar enhances waterlogging-induced adventitious root formation in cucumber by promoting auxin transport and signalling. *Plant Cell Environ* 43**,** 1545-1557.

Ruperti, B., Botton, A., Populin, F., Eccher, G., Brilli, M., Quaggiotti, S., Trevisan, S., Cainelli, N., Guarracino, P., Schievano, E., and Meggio, F. (2019). Flooding Responses on Grapevine: A Physiological, Transcriptional, and Metabolic Perspective. *Front Plant Sci* 10**,** 339.

Shang, P., Shen, B., Zeng, B., Bi, L., Qu, M., Zheng, Y., Ye, Y., Li, W., Zhou, X., Yang, X., Jiang, Y., and Zeng, B. (2023). Integrated Transcriptomic and Metabolomics Analysis of the Root Responses of Orchardgrass to Submergence Stress. *Int J Mol Sci* 24.

Wang, L., Li, D., Zhang, Y., Gao, Y., Yu, J., Wei, X., and Zhang, X. (2016). Tolerant and Susceptible Sesame Genotypes Reveal Waterlogging Stress Response Patterns. *PLOS ONE* 11**,** e0149912.

Wang, T., Zou, Q., Guo, Q., Yang, F., Wu, L., and Zhang, W. (2019). Widely Targeted Metabolomics Analysis Reveals the Effect of Flooding Stress on the Synthesis of Flavonoids in Chrysanthemum morifolium. *Molecules* 24.

Wang, X., He, Y., Zhang, C., Tian, Y.A., Lei, X., Li, D., Bai, S., Deng, X., and Lin, H. (2021a). Physiological and transcriptional responses of Phalaris arundinacea under waterlogging conditions. *J Plant Physiol* 261**,** 153428.

Wang, X., and Komatsu, S. (2016). Gel-Free/Label-Free Proteomic Analysis of Endoplasmic Reticulum Proteins in Soybean Root Tips under Flooding and Drought Stresses. *Journal of Proteome Research* 15**,** 2211-2227.

Wang, X., Li, F., Chen, Z., Yang, B., Komatsu, S., and Zhou, S. (2021b). Proteomic analysis reveals the effects of melatonin on soybean root tips under flooding stress. *J Proteomics* 232**,** 104064.

Wu, Y.S., and Yang, C.Y. (2020). Comprehensive Transcriptomic Analysis of Auxin Responses in Submerged Rice Coleoptile Growth. *Int J Mol Sci* 21.

Xiong, Q., Cao, C., Shen, T., Zhong, L., He, H., and Chen, X. (2019). Comprehensive metabolomic and proteomic analysis in biochemical metabolic pathways of rice spikes under drought and submergence stress. *Biochim Biophys Acta Proteins Proteom* 1867**,** 237-247.

Xu, J., Qiao, X., Tian, Z., Zhang, X., Zou, X., Cheng, Y., Lu, G., Zeng, L., Fu, G., Ding, X., and Lv, Y. (2018). Proteomic Analysis of Rapeseed Root Response to Waterlogging Stress. *Plants (Basel)* 7.

Yan, M., Xue, C., Xiong, Y., Meng, X., Li, B., Shen, R., and Lan, P. (2020). Proteomic dissection of the similar and different responses of wheat to drought, salinity and submergence during seed germination. *J Proteomics* 220**,** 103756.

Yeung, E., Van Veen, H., Vashisht, D., Sobral Paiva, A.L., Hummel, M., Rankenberg, T., Steffens, B., Steffen-Heins, A., Sauter, M., De Vries, M., Schuurink, R.C., Bazin, J., Bailey-Serres, J., Voesenek, L., and Sasidharan, R. (2018). A stress recovery signaling network for enhanced flooding tolerance in Arabidopsis thaliana. *Proc Natl Acad Sci U S A* 115**,** E6085-e6094.

Yu, F., Liang, K., Fang, T., Zhao, H., Han, X., Cai, M., and Qiu, F. (2019). A group VII ethylene response factor gene, ZmEREB180, coordinates waterlogging tolerance in maize seedlings. *Plant Biotechnol J* 17**,** 2286-2298.

Yu, F., Tan, Z., Fang, T., Tang, K., Liang, K., and Qiu, F. (2020). A Comprehensive Transcriptomics Analysis Reveals Long Non-Coding RNA to be Involved in the Key Metabolic Pathway in Response to Waterlogging Stress in Maize. *Genes (Basel)* 11.

Zaman, M.S.U., Malik, A.I., Erskine, W., and Kaur, P. (2019). Changes in gene expression during germination reveal pea genotypes with either "quiescence" or "escape" mechanisms of waterlogging tolerance. *Plant Cell Environ* 42**,** 245-258.

Zeng, B., Zhang, Y., Zhang, A., Qiao, D., Ren, J., Li, M., Cai, K., Zhang, J., and Huang, L. (2020). Transcriptome profiling of two Dactylis glomerata L. cultivars with different tolerance in response to submergence stress. *Phytochemistry* 175**,** 112378.

Zeng, N., Yang, Z., Zhang, Z., Hu, L., and Chen, L. (2019). Comparative Transcriptome Combined with Proteome Analyses Revealed Key Factors Involved in Alfalfa (Medicago sativa) Response to Waterlogging Stress. *Int J Mol Sci* 20.

Zhang, Y., Li, D., Zhou, R., Wang, X., Dossa, K., Wang, L., Zhang, Y., Yu, J., Gong, H., Zhang, X., and You, J. (2019). Transcriptome and metabolome analyses of two contrasting sesame genotypes reveal the crucial biological pathways involved in rapid adaptive response to salt stress. *BMC Plant Biology* 19**,** 66.

Zhang, Y., Zhao, P., Yue, S., Liu, M., Qiao, Y., Xu, S., Gu, R., Zhang, X., and Zhou, Y. (2021). New insights into physiological effects of anoxia under darkness on the iconic seagrass Zostera marina based on a combined analysis of transcriptomics and metabolomics. *Sci Total Environ* 768**,** 144717.

Zhao, N., Li, C., Yan, Y., Cao, W., Song, A., Wang, H., Chen, S., Jiang, J., and Chen, F. (2018). Comparative Transcriptome Analysis of Waterlogging-Sensitive and Waterlogging-Tolerant Chrysanthemum morifolium Cultivars under Waterlogging Stress and Reoxygenation Conditions. *Int J Mol Sci* 19.

Zhong, Z., Furuya, T., Ueno, K., Yamaguchi, H., Hitachi, K., Tsuchida, K., Tani, M., Tian, J., and Komatsu, S. (2020). Proteomic Analysis of Irradiation with Millimeter Waves on Soybean Growth under Flooding Conditions. *Int J Mol Sci* 21.

Zou, X., Jiang, Y., Liu, L., Zhang, Z., and Zheng, Y. (2010). Identification of transcriptome induced in roots of maize seedlings at the late stage of waterlogging. *BMC Plant Biology* 10**,** 189.
